# A transcriptome-based model of central memory CD4 T cell death in HIV infection

**DOI:** 10.1186/s12864-016-3308-8

**Published:** 2016-11-22

**Authors:** Gustavo Olvera-García, Tania Aguilar-García, Fany Gutiérrez-Jasso, Iván Imaz-Rosshandler, Claudia Rangel-Escareño, Lorena Orozco, Irma Aguilar-Delfín, Joel A. Vázquez-Pérez, Joaquín Zúñiga, Santiago Pérez-Patrigeon, Enrique Espinosa

**Affiliations:** 1 Department of Research in Immunology, Instituto Nacional de Enfermedades Respiratorias Ismael Cosío Villegas, Calzada de Tlalpan 4502, Mexico City, Mexico; 2 Computational Genomics Department, Instituto Nacional de Medicina Genómica, Periferico Sur 4809, Mexico City, Mexico; 3 Laboratory of Immunogenomics and Metabolic Diseases, Instituto Nacional de Medicina Genómica, Periferico Sur 4809, Mexico City, Mexico; 4 Department of Virology, Instituto Nacional de Enfermedades Respiratorias Ismael Cosío Villegas, Calzada de Tlalpan 4502, Mexico City, Mexico; 5 Infectious Immunopathogenesis Laboratory, Department of Infectious Diseases, Instituto Nacional de Ciencias Médicas y Nutrición Salvador Zubirán, Avenida Vasco de Quiroga 15, Mexico City, Mexico

**Keywords:** HIV, Immunologic Memory, Cell Cycle, Cell Death, CD4-Positive T-Lymphocytes, Transcriptome, Homeostasis

## Abstract

**Background:**

Human central memory CD4 T cells are characterized by their capacity of proliferation and differentiation into effector memory CD4 T cells. Homeostasis of central memory CD4 T cells is considered a key factor sustaining the asymptomatic stage of Human Immunodeficiency Virus type 1 (HIV-1) infection, while progression to acquired immunodeficiency syndrome is imputed to central memory CD4 T cells homeostatic failure. We investigated if central memory CD4 T cells from patients with HIV-1 infection have a gene expression profile impeding proliferation and survival, despite their activated state.

**Methods:**

Using gene expression microarrays, we analyzed mRNA expression patterns in naive, central memory, and effector memory CD4 T cells from healthy controls, and naive and central memory CD4 T cells from patients with HIV-1 infection. Differentially expressed genes, defined by Log_2_ Fold Change (FC) ≥ |0.5| and Log (odds) > 0, were used in pathway enrichment analyses.

**Results:**

Central memory CD4 T cells from patients and controls showed comparable expression of differentiation-related genes, ruling out an effector-like differentiation of central memory CD4 T cells in HIV infection. However, 210 genes were differentially expressed in central memory CD4 T cells from patients compared with those from controls. Expression of 75 of these genes was validated by semi quantitative RT-PCR, and independently reproduced enrichment results from this gene expression signature. The results of functional enrichment analysis indicated movement to cell cycle phases G1 and S (increased CCNE1, MKI67, IL12RB2, ADAM9, decreased FGF9, etc.), but also arrest in G2/M (increased CHK1, RBBP8, KIF11, etc.). Unexpectedly, the results also suggested decreased apoptosis (increased CSTA, NFKBIA, decreased RNASEL, etc.). Results also suggested increased IL-1β, IFN-γ, TNF, and RANTES (CCR5) activity upstream of the central memory CD4 T cells signature, consistent with the demonstrated milieu in HIV infection.

**Conclusions:**

Our findings support a model where progressive loss of central memory CD4 T cells in chronic HIV-1 infection is driven by increased cell cycle entry followed by mitotic arrest, leading to a non-apoptotic death pathway without actual proliferation, possibly contributing to increased turnover.

**Electronic supplementary material:**

The online version of this article (doi:10.1186/s12864-016-3308-8) contains supplementary material, which is available to authorized users.

## Background

Acute HIV infection depletes mucosal CD4 T cells, mainly effector memory (T_EM_) cells, rapidly and profoundly [1–3]. The ensuing chronic phase is largely asymptomatic, even though mucosal tissues are not replenished with T_EM_ cells [4]. Simian immunodeficiency virus (SIV) infection of rhesus macaques (an animal model of human HIV disease) shows that opportunistic control infection in the chronic phase is mediated by remnant mucosal T_EM_ cells supplied by the differentiation of central memory (T_CM_) cells in lymph nodes [5, 6]. Additionally, human T_CM_ cells are also characterized by their capacity of proliferation and differentiation into T_EM_ cells [7, 8]. Thus, homeostasis of T_CM_ cells is considered a key factor sustaining the asymptomatic stage of HIV infection, while progression to acquired immunodeficiency syndrome is attributed to homeostatic failure of T_CM_ cells [5, 6, 9–12].

It is unclear how this homeostatic equilibrium is lost during chronic infection. CD4 T cell maturation subpopulations (T_N_, T_CM_, and T_EM_) [7] are differentially affected by HIV infection [13, 14]; with T_EM_ cells being HIV’s main target [15]. T_CM_ cells can be infected in a lower proportion by HIV, which has led to propose that direct virion-mediated cytopathicity could gradually eliminate them, leading to poor homeostatic activity [6]. Nevertheless, direct cytopathicity by HIV [16] cannot completely explain CD4 T cell depletion during chronic infection [17–20], which suggests the participation of indirect pathogenic mechanisms, particularly chronic activation [12, 21]. Additionally, CD4 T cells from patients with HIV could be intrinsically altered, as suggested by the limited proportion of HIV-infected patients recovering their pre-infection CD4 T cells counts under virus-controlling antiretroviral therapy [22]. In this regard, we have found intrinsic dysfunctions in activated T_CM_ cells from HIV-infected patients, as a lowered IL-2 response and CD40L induction after T cell receptor (TCR)-mediated stimulation [23, 24], which could decrease their proliferative, differentiation, and survival capacities.

In order to determine if circulating T_CM_ cells from HIV-infected patients have a transcriptome consistent with activation, but simultaneously with altered capacities to divide and survive, we compared the *ex-vivo* messenger Ribonucleic acid (mRNA) whole-genome expression patterns of CD4 T naive (T_N_) and T_CM_ cells from HIV^+^ patients with T_N_, T_CM_, and T_EM_ cells from healthy controls. We found a T_CM_ cell signature in HIV-1 infection suggesting that the loss of this subpopulation may be driven by increased cell cycle entry followed by mitotic arrest possibly leading to cell death in a non-senescent or effector-like state.

## Methods

### Participants

This study was approved by the boards of Instituto Nacional de Enfermedades Respiratorias Ismael Cosío Villegas (reference number B29-11), and Instituto Nacional de Ciencias Médicas y Nutrición Salvador Zubirán (reference number 1403). All patients signed written informed consent according with the Helsinki Protocol. Blood samples were obtained from 9 HIV¯ controls, and 6 HIV^+^ patients. Patients had median 480 CD4 T cells/μL blood (range 330–757), and median 121 563 HIV-ribonucleic acid (RNA) copies/mL-blood (23 883–41 2584). Among them, patients providing T_CM_ cells had viral loads of 23 883, 81 834 and 107 732 HIV RNA copies/mL-blood, and CD4 T cell counts of 439, 473 and 491 CD4 T cells/μL blood, respectively. Relative telomere length was determined in samples from ten additional HIV¯ controls, and ten additional HIV^+^patients with median 628 CD4 T cells/ μL-blood (194–1 128) and median 485 882 HIV-RNA copies/mL-blood (3 870–3 500 000). Patients were antiretroviral therapy-naive, free of opportunistic infections and malignancies, and were not taking any immunomodulatory drugs.

### Isolation of CD4 T cell subpopulations

Peripheral blood mononuclear cells (PBMCs) were purified from 50 to 60 mL of peripheral blood by sedimentation on Lymphoprep (Fresenius Kabi Norge, Oslo, Norway). CD4 T_N_ (CD45RA^+^ CCR7^+^), T_CM_ (CD45RA¯ CCR7^+^) and T_EM_ (CD45RA¯ CCR7¯) cells were purified from PBMCs using immunomagnetic beads (Miltenyi Biotec, Bergisch Gladbach, Germany).

Subpopulation purity was determined according to the expression of CD4, CD45RA and CCR7, using anti-CD4-APC-Cy7, anti-CD45RA-APC (BD Biosciences, San José, CA, USA), and anti-CCR7-PE (Miltenyi Biotec) fluorochrome-conjugated antibodies (See Additional file 1). Cells were analyzed in a FACSCanto II flow cytometer (BD Biosciences). Cells with purity >90% were used. Membrane CD38 was detected with an anti-CD38-biotin (Miltenyi Biotec) plus streptavidin PerCp-Cy5.5 (Biolegend, San Diego, CA, USA).

### RNA extraction and microarray analysis

Total RNA was obtained from three T_N_, three T_CM_, and three T_EM_ CD4 T cell samples from healthy controls, and three T_N_ and three T_CM_ CD4 T cell samples from HIV^+^ patients, using RNeasy Mini Kit (Qiagen, Venlo, Netherlands). Each RNA sample proceeded from a different subject. Scarcity of patients’ T_EM_ cells precluded obtaining sufficient RNA.

Microarray gene expression analysis used equimolar concentrations of total RNA from T cell subpopulations. Complementary deoxyribonucleic acid (cDNA) synthesis, amplification, and gene expression profiling were performed according to the manufacturer’s instructions (Affymetrix WT Sense Target labeling assay manual, California, USA). Labeled DNA was added to hybridization cocktail and injected into the array (GeneChip Human Gene 1.0 ST Array, Affymetrix). Washing and staining steps were performed in the GeneChip Fluidics Station 450 (Affymetrix). Probe arrays were scanned using a GeneChip Scanner 3000 7G (Affymetrix). Data were deposited in GEO, series record GSE73968.

Background correction and normalization were performed with Robust Multiarray Average Method (RMA) [25] using Bioconductor package [26] of R [27]. A Principal component analysis (PCA) of normalized signals from all genes in each microarray was performed using R [27].

Modeling gene expression was performed using linear models of Limma package [28]. The B-statistic was used as significant measure to define differentially expressed genes. This statistic is computed as the posterior odds of differential expression. It is reformulated in terms of a moderated t-statistic in which posterior residual standard deviations are used in place of ordinary standard deviations. Essentially, the B-statistic compromises between individual gene variance estimates and a single variance estimate for all genes. The probabilities are transformed to a scale that goes from –Inf to Inf using log odds. The B-statistic is analogous to the adjusted *p*-value, which addresses statistical significance for multiple comparisons. Here, genes with Log 2 Fold Change (FC) ≥ |0.5| and Log odds > 0 were considered as differentially expressed. Limma statistics such as adjusted *p*-value and the B statistic can be seen in Additional file 2. FDR Benjamini Hochberg multiple testing correction [29] was applied to control the number of false positives. Both B statistic and adjusted *p*-value showed consistency across differentially expressed genes. Unsupervised 2-way hierarchical clustering analysis of gene expression data was performed using Euclidian distance and average linkage with gplots [30] of R [27]. Venn diagrams were made with Venny 2.0.2 [31].

Functional enrichment analyses were performed with Data Base for Annotation, Visualization and Integrated Discovery (DAVID) [32, 33], Gen Set Enrichment Analysis (GSEA) [34] and Ingenuity Pathway Analysis (IPA, QIAGEN Redwood City, CA, USA). DAVID uses a Fisher Exact test in order to determine gene-enrichment in annotation terms. A gene set is enriched when the proportion of genes in a list that falls into an annotation term differs from the background model. The EASE score is a modified Fisher exact *p*-value. Basically, if *n* is the number of genes in the list that falls into a given annotation term, *n-1* is used to compute the *p*-value [32, 33]. Gene set enrichment methods also implement strategies for addressing the issue of multiple testing hypotheses. GSEA uses a ranking procedure to produce a gene list from the full expression matrix. This is done by computing an Enrichment Score (ES(S)). It controls the ratio of false positives to the total number of gene sets attaining a fixed level of significance using FDR [34]. IPA assesses enrichment (i. e. biological functions that could be increased or decreased given the observed gene expression patterns) using a Fisher exact *p*-value. Additionally, it computes a Z score that allows inferring upstream transcriptional regulators and expectable enriched functions, based on statistical significance by comparing the match between observed and predicted up/down regulation patterns. The null model is referred as activation Z-score [35]. Predicted regulation patterns are based on previously reported causal relationships between relevant genes and functions [35].

### Semi-quantitative real-time PCR

We used *B2M*, *GAPDH*, *POLR2A*, and *TBP* as reference genes to normalize expression. RNA proceeded from the samples used for microarray analysis. cDNA was synthesized from ~100 ng total RNA with Transcriptor First Strand cDNA Synthesis Kit (Roche Applied Science, Mannheim, Germany), using random hexamers and performing one cycle of 10 min 25 °C; 30 min 55 °C, and 5 min 85 °C. cDNA was stored at −20 °C until use. PCR amplifications were performed by high-throughput gene expression analysis using DNA binding dye Evagreen (SsoFast MasterMix, Biorad, California, USA) for product detection, and specific primers for each gene (DELTAgene Assays, Fluidigm Corporation, California, USA). Specific target pre-amplification of each cDNA and a cleanup step were performed as described elsewhere [36].

We performed semiquantitative RT-PCR using Fast Gene Expression Analysis with EvaGreen (Biorad), following the Biomark System Protocol (Fluidigm Corporation, California, USA). Assay mixes (100 μM of each pair of primers, 2X Assay Loading Reagent, and 1X TE buffer), sample mixes (pre-amplified cDNA, 2X SsoFast MasterMix (BioRad), and 20X DNA Binding Dye Sample Loading Reagent (Fluidigm), were loaded into a 96.96 Dynamic Array (Fluidigm), using the IFC Controller HX (Fluidigm), and were then transferred to a BioMark HD device (Fluidigm) for the PCR cycles (40 min 70 °C, 30s 60 °C; 60s 95 °C, then 30 cycles of 5 s 96 °C, 60s 60 °C). Melting curves were determined at the 60 to 95 °C rise, with a temperature change rate of 1 °C/3 s. Ct values were obtained with Fluidigm Real-Time PCR Analysis Version 4.1.3 software (Fluidigm).

Only Ct values <30 and amplicons with only 1 melting curve were used. Geometric means of four reference genes were used to normalize expression data [37]. Relative expression was calculated as ΔΔCt. Expression of each gene was determined with six technical replicates per sample. Normality was verified using Kolmogorov-Smirnov test, which quantifies the distance between the empirical distribution of the sample and the cumulative distribution of the reference distribution, which in this case is assumed to be normal. Group differences were analyzed with Student’s t test. Data management and statistics were done with Reshape [38] and fBasics [39] packages of R [27].

### Relative telomere length

Telomere PNA kit/FITC (Dako, São Paulo, Brazil) was used following the manufacturer’s instructions, including thymocytes from 6-week old mice as reference for normalization. Briefly, samples were prepared by mixing 10^6^ mouse thymocytes and 10^6^ T_CM_ cells. The mixture was distributed into four tubes. 150 μl of FITC-labeled peptide nucleic acid (PNA) probe solution was added into two tubes while 150 μl of unlabeled PNA probe solution was added into the other two. Samples were hybridized in a pre-warmed heating block (TB2 Thermoblock, Biometra, Göttingen, Germany) set at 82 °C, 10 min, and left overnight at room temperature. Samples were washed twice. Between washing steps, samples were heated to 40 °C in a pre-warmed TB2 Thermoblock (Biometra) for 10 min. Samples were resuspended in 250 μL of DNA staining solution (1X), and stored overnight at 4 °C, away of light. Then, samples were analyzed by flow cytometry in a FACSCanto II (BD Biosciences).

## Results

### T_CM_ cells from HIV^+^ patients are not more differentiated and are not more senescent

**Fig. 1:**
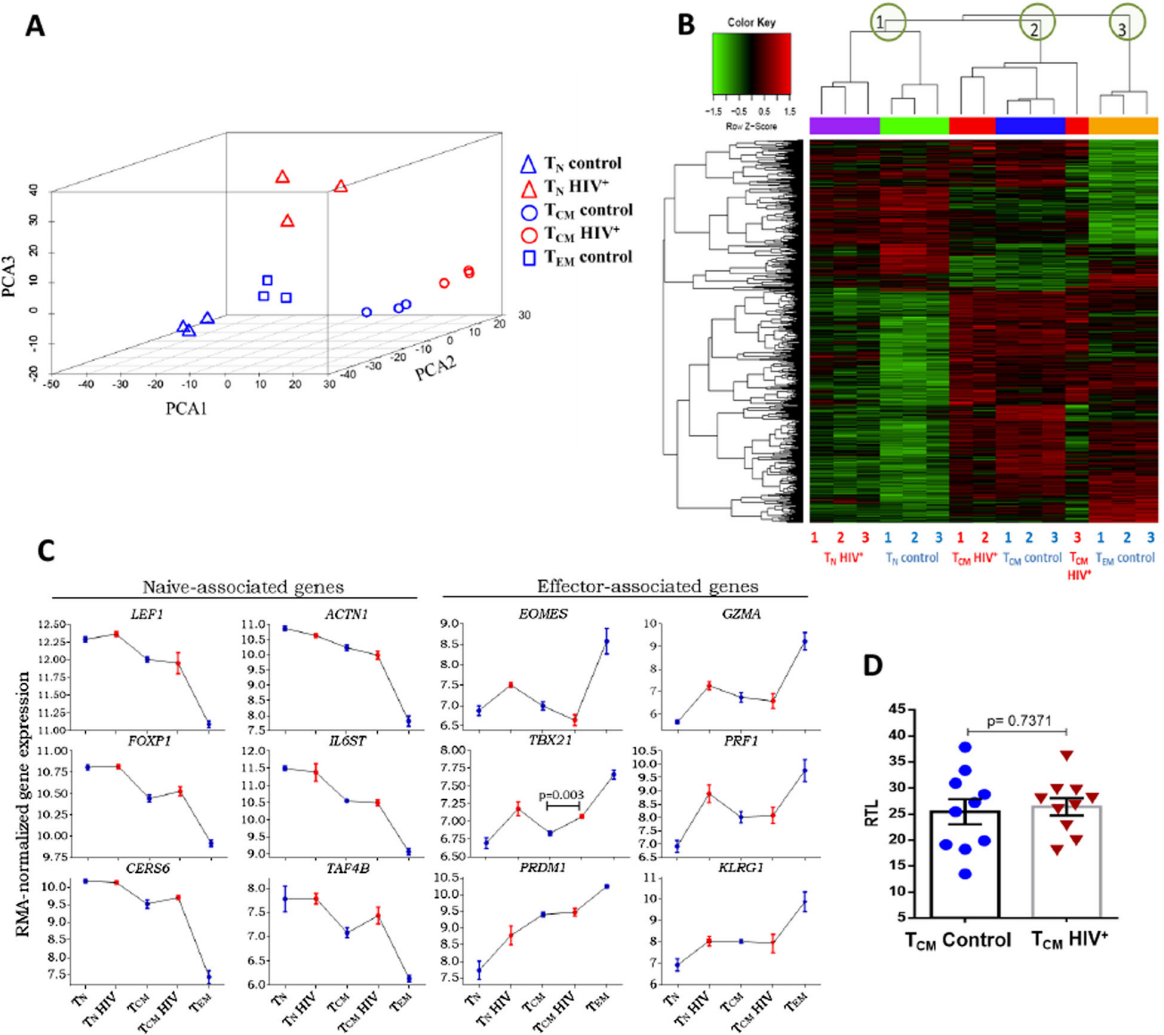
Gene expression does not support greater differentiation or senescence of T_CM_ cells from HIV^+^ patients. **a** Principal component analysis of the entire microarray data set of each subpopulations from HIV^+^ patients (*red triangles* and *red circles*) and controls (*blue triangles*, *blue circles* and *blue squares*). The first three principal components are shown, accounting for 55% of variance in a three dimensional plot. **b** Heat map resulting from hierarchical clustering of genes related with normal differentiation (pairwise comparisons between not infected subpopulations). Each row represents a differentially expressed gene. Each column represents each independent sample. The unsupervised two-way hierarchical clustering is shown as a dendrogram for genes (*left*), and a dendrogram for samples (*top*). In the upper dendrogram (samples) the independent resulting nodes, each one corresponding to a maturation subpopulation, is encircled in green. **c** Sequential downregulation of selected naïve-associated genes, and sequential upregulation of selected effector-associated genes when samples are arranged according to the linear differentiation model. Data are represented as means ± 1 SEM of three donors (*blue*) and three patients (*red*). TBX21 expression difference between T_CM_ and T_CM_ HIV was analyzed with Student’s t test. **d** Relative telomere length of central memory CD4 T cells (T_CM_) from HIV^+^ patients (*red triangles*) and controls (*blue circles*), Student’s t-test was used to compare groups. We were unable to obtain sufficient RNA from T_EM_ cells from patients due to their small number

Unsupervised principal component analysis of normalized whole genome expression data segregated samples of each maturation subpopulation, and further separated samples originating from persons with HIV and samples from controls (Fig. 1a). Thus, phenotype-based classification of differentiation subpopulations [7, 8] reliably reflected distinct gene expression programs, as previously reported [40–42], which were altered by HIV infection.

**Fig. 2:**
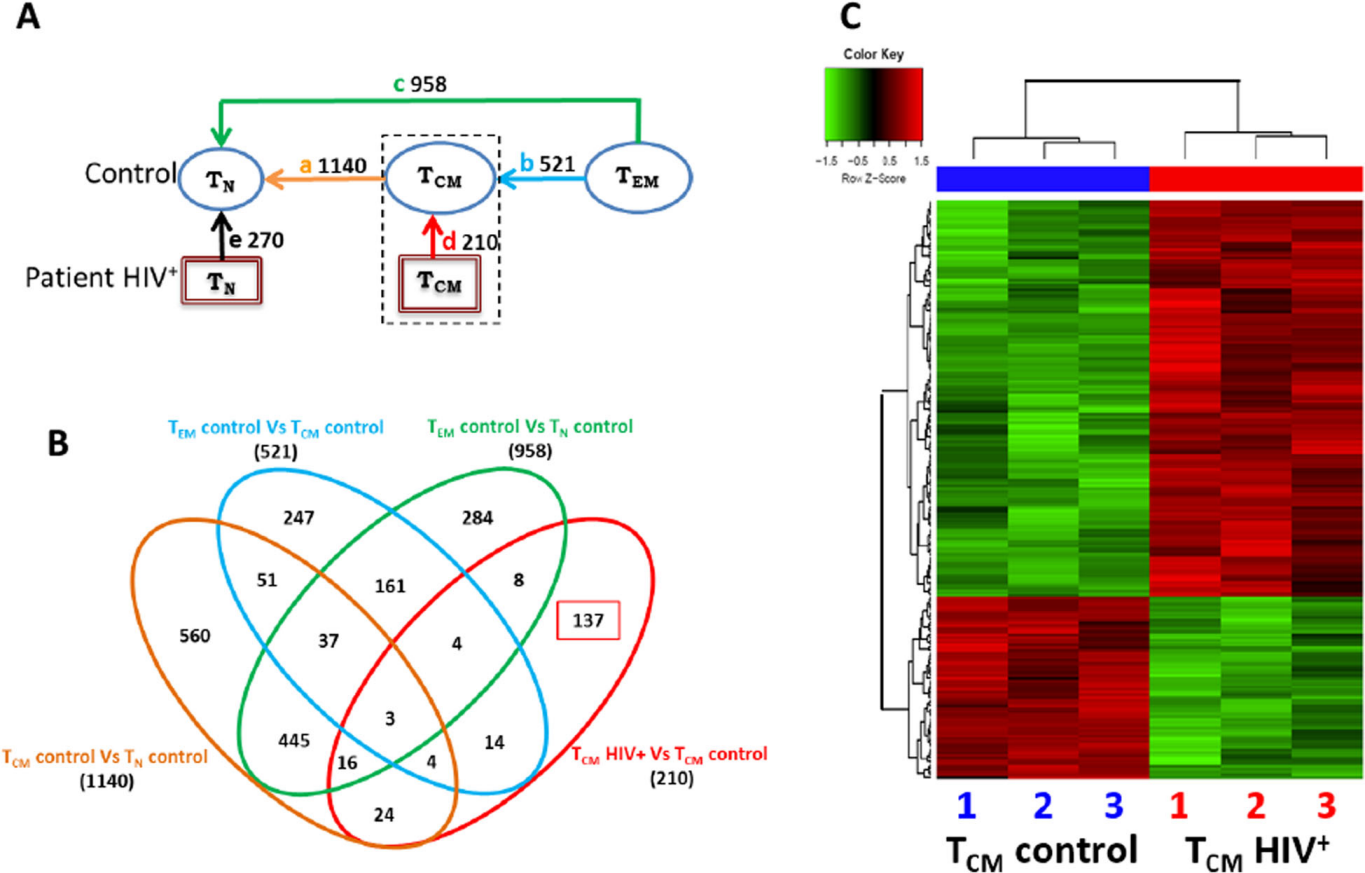
Unique T_CM_ cell signature in HIV infection. Differential expression was defined as Log_2_ of fold change (Log _FC_) ≥ |0.5|, and Log (odds) > 0. **a** Pairwise comparisons of samples of CD4 T cells subpopulations from HIV^+^ and HIV¯ groups indicated by arrows **a** and **d**. Number of genes differentially expressed in each comparison are shown. Blue circles, controls’ samples; red squares, HIV^+^ patients’ samples. **b** Venn diagram of sets of differentially expressed genes. Each pairwise comparison is depicted by a colored oval. The number of differentially expressed genes found in more than one comparison appear in the intersections. **c** Heat map displaying a two-way unsupervised hierarchical clustering of 210 differentially expressed gens distinguishing HIV^+^ patients’ T_CM_ cells (*red bar*) and controls’ T_CM_ cells (*blue bar*), grouped in dendrograms. Each column represents an independent sample (biological replica) of each subpopulation, numbered 1 to 3. Each row corresponds to a differentially expressed gene

We asked if differential gene expression by patients’ and controls’ T_CM_ cells reflected greater differentiation of patients’ cells (towards effector stages) [23, 24]. Using the criteria defined in methods (Log_2_FC ≥ |0.5| and Log (odds) > 0) we looked in the whole transcriptome for all differentially expressed genes in the following pair-wise comparisons of CD4 T cell subpopulations from controls: T_CM_ vs. T_N_, T_EM_ vs. T_CM,_ and T_EM_ vs. T_N_ (arrows a, b and c in Fig. 2a). The resulting 1858 differentially expressed genes are subsequently referred to as differentiation-related genes (corresponding to subpopulations in distinct stages of differentiation). We performed an unsupervised 2-way hierarchical clustering analysis of these 1858 differentiation-related genes (Fig. 1b, and Additional file 3). T_N_ and T_CM_ cells from patients grouped with their counterparts from controls (Fig. 1b). Samples of a same subpopulation were assigned to a same node (green circles 1, 2, and 3 on Fig. 1b), regardless of their HIV status. The expression of the differentiation-related genes progressively decreased or increased in the order of linear differentiation (T_N_ → T_CM_ → T_EM_ ), agreeing with previous reports [40–43] (Fig. 1c). For instance, *LEF1, ACTN1, FOXP1*, *IL6ST* and *CERS6* reportedly undergoing down-regulation in naive T cells after antigen recognition and differentiation [44–48], along with TAF4B, appeared progressively down regulated when samples were ordered according to the linear model of peripheral differentiation (Fig. 1c, left panel). These changes agree with previous reports [42]. Conversely, differentiation and effector function-associated transcripts, like *EOMES*, *TBX21* (t-bet), *PRDM1* (Blimp-1) [49, 50], GZMA and PRF1 [51, 52], were gradually increased in the same order (Fig. 1c right panel). A same pattern was followed by the expression of *KLRG1,*an indicator of replicative senescence [53, 54] (Fig. 1c). *TBX21* (t-bet) was the only gene with increased expression in T_CM_ cells from patients, compared with controls (*p* = 0.003), which, along with the increased expression of IL12R e IL18R, suggests a Th1-skewed response driven by HIV infection. A Th1-skewed response was also predicted by Ingenuity Canonical Pathway analysis (See Additional file 4). Thus, T_CM_ cells from patients did not seem to be more differentiated than their counterparts from controls, but appeared polarized to Th1 functions.

We then asked if patients’ T_CM_ cells had a longer replicative history, which would entail a shortening of telomeres. We did not find any difference in relative telomere length between T_CM_ cells from patients and controls (*p* = 0.737, Fig. 1d), agreeing with KLRG1 expression [53, 54], and suggesting that they are not in a more senescent state.

### T_CM_ gene expression signature in HIV infection

Having ruled out a greater differentiation of patients’ T_CM_ cells, we investigated if the gene expression signature of these cells revealed a functional state that could explain loss of homeostatic capacity. Using the criteria defined in methods (Log_2_FC ≥ |0.5| and Log (odds) > 0), we looked for genes that were differentially expressed by T_CM_ cells from HIV^+^ patient and T_CM_ from controls. We found a total of 210 differentially expressed genes. We refer to this 210- gene list as the gene expression signature of T_CM_ cells in HIV infection (See Fig. 2a arrow d, b red oval and Additional file 2). This gene expression signature was obtained from the transcriptome independently of the list of 1858 differentiation-related genes. Among these 210 differentially expressed genes, 137 were absent in all other pairwise comparison (Fig. 2a, b). Hierarchical clustering analysis showed clear and consistent differences in the relative expression of these 210 genes between patients and controls (Fig. 2c). Of note, biological replicates were very homogeneous.

We analyzed the HIV T_CM_ signature with the enrichment analysis tools IPA, GSEA, and DAVID. These different analyses consistently yielded four general functional categories that were modified in T_CM_ cells from patients: cell cycle, DNA damage and repair, apoptosis, and immune responses (Table 1). Notably, the 137 genes uniquely distinguishing T_CM_ cells from patients and controls (Fig. 2b red oval) sufficed to yield the same four functional categories when analyzed with DAVID and IPA. This suggests that the enriched functions largely depend on the T_CM_ cell signature. GSEA rendered a larger set of altered immune functions, likely because it uses data from the entire microarray, and because it detects more modest changes when the members of a function or pathway show a strong correlation [34] (Table 1). GSEA identified Toll-like receptors (TLR), type I interferons, IL1, and NLRs signaling, plus NFκB activation, all of them related to an inflammatory milieu. Independently, Ingenuity Upstream Regulator Analysis [35] assigned the greatest z-scores and the most significant *p*-values to the activity of IL-1B, TNF, NFκB complex, and CCL5, as possible upstream molecules eliciting the expression changes constituting the T_CM_ signature (See Additional file 5, upstream analysis). Cell cycle, DNA damage and repair, and apoptosis (greatly related functions [55, 56]) appeared consistently in the output of all enrichment analysis tools (Table 1). There were several functional categories closely related with G0/G1/S transition and G2/M checkpoints in the output of GSEA analysis. IPA, which weighs its predictions, displayed increased proliferation and cell survival, decreased apoptosis and decreased cell death. In contrast, IPA’s output simultaneously indicated an increase in cytostasis, movement to interphase, and a decrease in mitosis (Table 1).

**Table 1:** Enriched categories of functions according to TCM gene expression signature in HIV infection

General category	DAVID		GSEA	IPA		
	EASE < 0.05	Number of genes	FDR < 0.05, *p* < 0.001	*p* < 0.01	Number of genes	Prediction sense
Cell cycle	Cell cycle	15	DNA replication	Proliferation of tumor cell lines	31	Positive
	Cell division	10	Cell cycle	Proliferation of cells	53	Positive
	mitosis	9	Mitotic M/G1	Cytostasis	6	Positive
			G1/S transition	Cytostasis od tumor cell lines	5	Positive
			Cell cycle check points	Interphase of tumor cell lines	11	Positive
			Cyclin E associated event during G1/S transition	Cell survival	28	Positive
			Assembly of pre-replicative complex	Interphase	13	Positive
			G0 and early G1	Mitosis	10	Negative
			G2/M check points			
DNA damage or repair	p53 signaling pathway	5	p53 dependent G1 DNA damage response			
	ATM signal pathway	3				
	Cell cycle checkpoints	5				
Apoptosis	Apoptosis	13		Apoptosis of tumor cell lines	32	Negative
				Cell death of cancer cells	6	Negative
				Apoptosis of cervical cancer cell lines	10	Negative
Immune responses			Toll endogenous pathway	Synthesis of reactive oxygen species	7	Positive
			IL1 signaling			
			IFN-alpha/beta signaling			
			Chemokine receptors bind chemokines			
			NOD like receptors signaling			
			NFKB activation by IKKS complex			
			Myd88 cascade			
			TLR4 signaling			
			IL12 pathway			
			FOXO Pathway			

Enriched categories of functions according to differential expression of 210 genes in T_CM_ cells from HIV^+^ patients and controls. DAVID and IPA tools show in a column the number of genes supporting each prediction. EASE Score is the *P*-Value of a modified Fisher Exact test of the significance of gene enrichment in a gen-set. FDR: False discovery rate. In IPA, the sign indicates if the function would be up-regulated (positive) or down-regulated (negative)

**Fig. 3:**
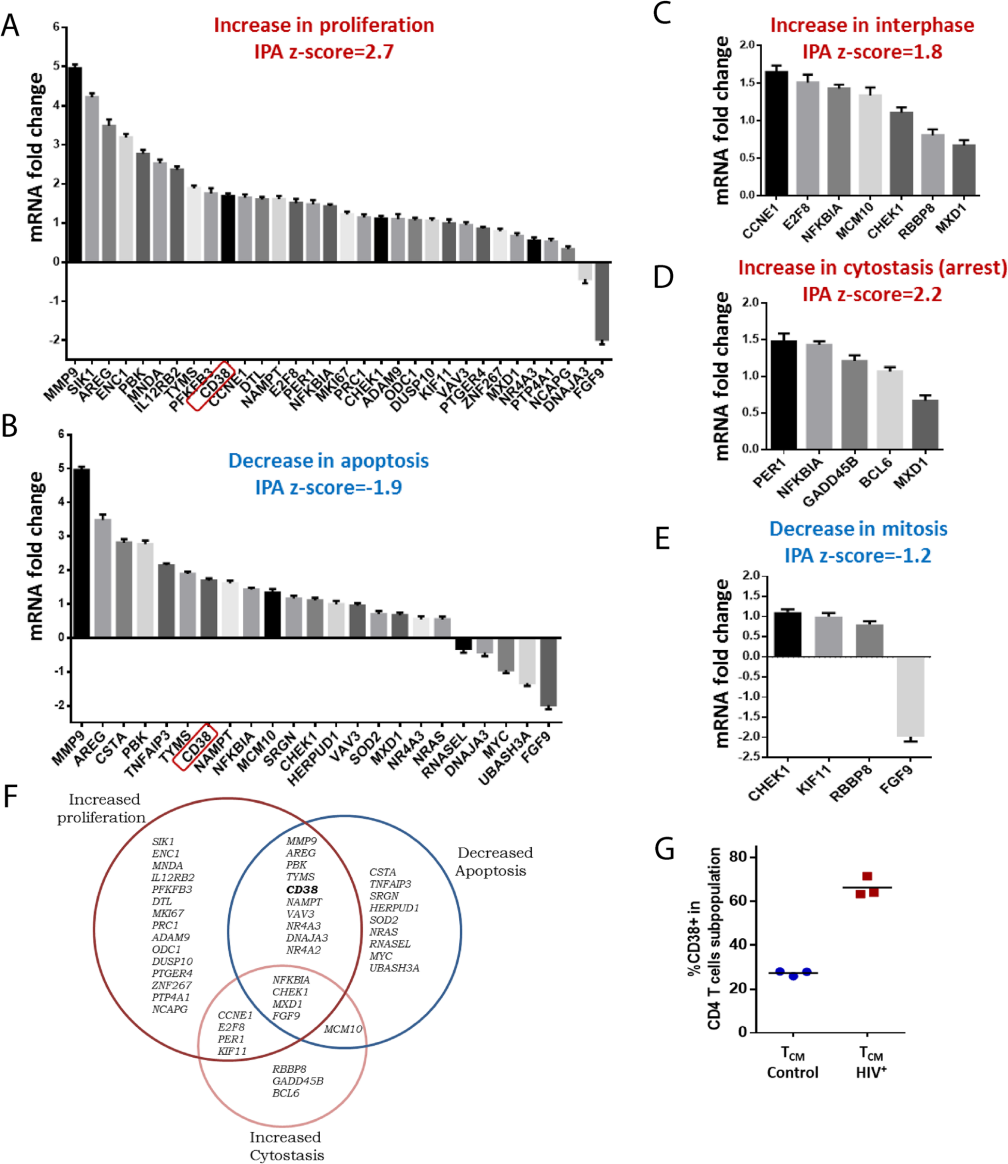
Cell cycle impairments are predicted using RT-PCR-validated genes from the signature. Each graph (**a** to **e**) represents the group of validated genes by RT-PCR associated with an increased enriched function (*red title* and *positive z-score*) or decreased enriched function (*blue title* and *negative z-core*). **f** Venn diagram depicting the differentially expressed genes within sets corresponding to three IPA predictions. Intersections correspond to genes appearing in more than one prediction. **g** Frequency of surface expression of CD38 on T_CM_ cells from two groups, corresponding to gene expression results

We re-analyzed mRNA expression by RT-PCR of 91 genes of the HIV T_CM_ signature that were associated with enriched functions, and *B2M*, *GAPDH*, *POLR2A*, and *TBP* as reference genes. This analysis validated 75 genes (82%) of the signature (See Additional file 6). Five genes failed amplification, and 11 were not differentially expressed when assessed by RT-PCR. The expression of reference genes did not differ between samples (See Additional file 7). An analysis with IPA using only the 75 validated genes yielded the same enriched functions and pathways as microarray data (Fig. 3a, b, c, d, e, and Additional file 8). While some genes were related with more than one function, many were related exclusively with one function (Fig. 3f), supporting an unambiguous prediction. Finally, analyzing the T_CM_ cells used for microarray and RT-PCR analyses, we found that surface expression of the CD38 protein was more frequent among T_CM_ cells from patients than among those from controls, consistent with mRNA results (Fig. 3g).

### A model of T_CM_ cell death in HIV infection

**Fig. 4:**
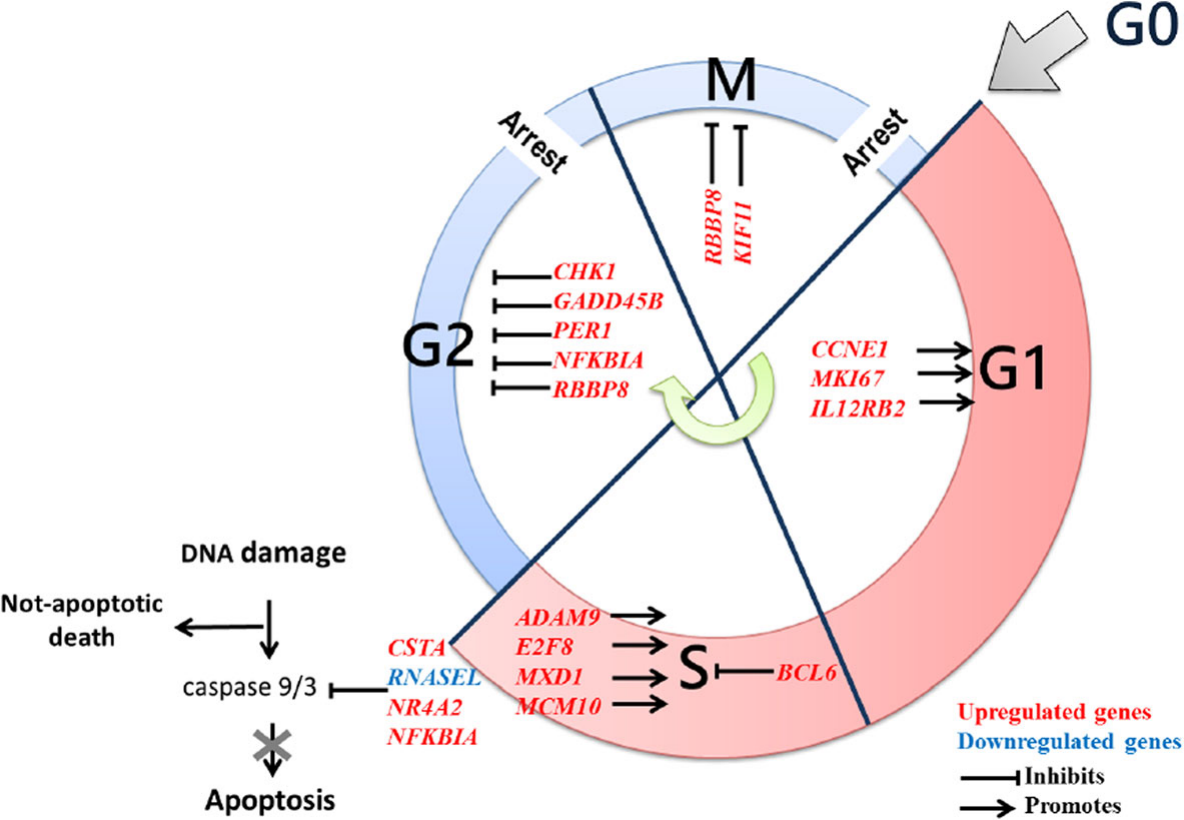
Allocation of HIV-related T_CM_ signature genes in the cell cycle phases they regulate. These genes suggest a promoted progress from G0 to S, followed by arrest in G2/M cell cycle phases, and decreased apoptosis

Since the predictions of increased proliferation and increased cytostasis were incompatible, and the prediction of reduced apoptosis did not agree with previous evidence [13, 57–60], we took into account that enrichment tools base their predictions on a broad set of previous findings, ranging from very particular to very general ones. Accordingly, we investigated if the predictions were based on more demarcated processes, and if these processes were compatible. With this purpose, we reviewed the references supporting IPA predictions, allocating the indicated genes to the cell cycle phase that they regulated. We found that genes in all the predictions could be assigned to particular phases in the cell cycle, and implied no conflicts (Fig. 4), with the exception of some proliferation-predicting genes, which did not relate with cell cycle in the supporting evidence.

The HIV T_CM_ signature is compatible with an enhanced progression from G0 to S phase (CCNE1 [61, 62], MKI67 [63, 64], IL12RB2 [65, 66], ADAM9 [67], E2F8 [68], MXD1 [69], MCM10 [70], BCL6 [71–73]), but not with progression to later stages. Simultaneously, the expression patterns of other genes suggest arrest in G2/M (CHK1 [74], GADD45B [75], PER1 [76], NFKBIA [77], RBBP8 [78], KIF11 [79]). For instance, accumulation of *CCNE1* is necessary for G1 → S transition, but its overexpression is associated with chromosome destabilization and DNA damage [61, 62]. In turn, CHK1 expression is required for cycle arrest in G2 following DNA damage [74]. These observations suggest that the cell processes expectable from the HIV T_CM_ signature can be integrated within the cell cycle, and they indicate increased cycling up to S phase, followed by arrest in G2/M. Remarkably, since cycle arrest leads to cell death [55, 56], it was unexpected to find that the expression patterns of CSTA [80], RNASEL [81] NR4A2 [82] and NFKBIA [77] predicted an inhibition of caspase-3 mediated apoptosis (Fig. 4).

## Discussion

We identified and validated a gene expression signature of T_CM_ cells in the context of chronic HIV infection that led us to a model of T_CM_ cell death in HIV infection, also supported by additional observations and previous reports.

It could be argued that a model based on mRNA presence, but not demonstrating the presence of encoded proteins [83], is unfounded. In this regard, recent studies and reassessments found that mRNA changes explained 87 to 92% of corresponding protein changes [84, 85], as found in mouse dendritic cells responding to lipopolysaccharide [86]. Further, 81% of protein levels were explained by mRNA levels using a large scale proteome and mRNA analysis in NIH3T3 cells [87]. Therefore, we consider that we have evidence to propose a model of T_CM_ cell death in HIV infection.

Our interest in intrinsic T_CM_ cell alterations in patients with HIV originated from studies showing their importance in CD4 T cell homeostasis under HIV infection [10, 12, 57, 88–90]. Our previous studies on activated (CD38^+^) T_CM_ cells, particularly those from HIV-infected patients, showed IFN-γ-skewed cytokine responses that were un-connected to CD40L induction, along with a lowered IL-2 production [23, 24]. Given this functionality, T_CM_ cells seemed differentiated towards an effector fate [40, 42, 43, 91, 92]. However, in the present study we found that T_CM_ cells’ gene expression profile was incompatible with the T_EM_ maturation category. Additionally, we found no decrease in relative telomere length (RTL) of patients’ T_CM_ cells, which is expectable of cells with a longer replicative history [7, 8], such as T_EM_- cells. Moreover, KLRG1 expression, which is proportional to replicative history [53], was similar in T_CM_ cells from patients and controls, but greatly increased in more differentiated T_EM_ cells. These findings support that HIV infection was not associated with an enhanced T_CM_ cell differentiation. Nevertheless, T_CM_ patients’ cells showed an increased expression of the Th1-associated transcription factor T-bet [49, 93], suggesting that our previous findings were attributable to Th1 polarization rather than differentiation.

The homogeneity and consistency of the T_CM_ signature in HIV infection (Fig. 2c) contrasts with the great differences between patients’ viral loads (23883, 81834 and 107732 HIV RNA copies/mL-blood). This may seem more important considering that even transient changes in viral load can greatly influence gene expression in total CD4 T cells [94]. However, patients’ CD4 T cell counts, the strongest predictor of subsequent disease progression [95] are close (439, 473 and 491 CD4 T cells/mm^3^ blood, respectively), and lie above the threshold for the occurrence of most opportunistic infections (200 cells/mm3 blood [95]). This could suggest that the T_CM_ gene expression signature is not dependent of the magnitude of viral replication during the chronic phase of infection, but it could rather be related with irreversible events from the initial phase of infection and/or with the magnitude of circulating CD4 T cell loss in the chronic phase.

A thorough pathway enrichment analysis of the HIV T_CM_ signature suggested enhanced cell cycle entry and proliferation. Under closer inspection, however, we observed that proliferation-predicting genes corresponded to functions upstream of S phase. Conversely, this analysis also predicted cell cycle arrest due to functions occurring in G2 or M. If cell cycle promotion and arrest occur in a same T_CM_ cell, cycling would not imply cell division [58], since G2/M arrest would lead to death [96]. Additionally, overexpression of DNA damage and repair-related genes by patients’ T_CM_ cells are consistent with a failed division after S phase.

Our model could integrate partial observations from previous studies on cell cycle in HIV infection, providing a wider view of the fate of T_CM_ cells. In a previous study, CD4 T cells from patients with HIV that were *ex vivo* in S phase (mostly T_CM_) would die after in vitro stimulation more frequently than CD8 T cells [58]. However, patients’ cells were not compared with CD4 T cells from HIV¯ controls. Contrastingly, our comparison with cells from controls, and our characterization of CD4 T cell subpopulations, suggested that cycle-related death was due to HIV infection, and involved predominantly T_CM_ cells.

Another group reported arrest in G1 based on the accumulation of cycling cells in G1 among total circulating CD4 T cells [97]. However, since T_CM_ cells comprise only about 25% of circulating CD4 T cells [42], they were possibly not well represented in that study. Nevertheless, an increased proportion of Ki67^+^ cells in the G1 phase provided evidence that CD4 T cells more frequently entered cell cycle in HIV infection, as previously demonstrated [98, 99]. Since dead cells are readily removed from blood [100], it is possible that the reported ex vivo increase of CD4 T cells in G1 was the result of rapid removal from blood of cells that died in a further phase. Our findings were consistent with a promoted entry into cell cycle (Fig. 4), and notably, they suggested that arrest occurred in the later phases G2 and/or M.

Our results could imply that incorporation of nucleotide analogues by T_CM_ cells from patients with HIV could reflect entrance to a fatal cycle, rather than proliferation [101, 102]. Evidence of division and viability will be required to dismiss this possibility. In our model, T_CM_ cell turnover reflects to some extent, death, and not actual proliferation. Accordingly, we found no difference in telomere length between T_CM_ cells from controls and patients.

An analogous implication pertains to apoptosis. Previous studies have reported increased apoptosis in total [58, 103, 104] and in T_CM_ [58, 105] CD4 T cells from patients with HIV, which is an explanation of CD4 T cell loss in chronic infection [106]. These and other studies [13, 57–60] inferred apoptosis by demonstrating Annexin V binding to viable cells; however, it has been demonstrated that Annexin V binding can be increased in other cell death pathways [107–110]. In this regard, we found that HIV infection altered the expression of a considerable number of genes that indicated that apoptosis would not be favored. A possible explanation of this discordance with previous studies could be that different cell death pathways may coexist with caspase 3-mediated T_CM_ cell death in HIV infection, as suggested by other studies [107, 111]. Also, T_CM_ subpopulation is heterogeneous [10, 112], and cells under apoptosis might not be reflected in microarray data. Therefore, we propose that an additional programmed cell death pathway may be in involved in T_CM_ cell death after cell cycle arrest. A likely pathway is pyroptosis, an inflammatory programmed cell death pathway driven by pro-inflammatory signals, such as bacterial lipopolysaccharide and IL-1β [113]. This pathway has been suggested by previous reports [111, 114], and agrees with the presence in blood of triggers of pyroptosis as bacterial lipopolysaccharide (LPS) [115] and IL-1β [116] in HIV infection. Additionally, increased concentrations of IL-18, a cytokine liberated during pyroptosis, has been found in the blood of patients with HIV [117].

## Conclusions

In summary, we propose a model of CD4 T_CM_ cell death in chronic HIV infection based on a gene expression signature unique to this subpopulation. According to it, CD4 T_CM_ cell loss in HIV infection may be driven in vivo by increased cell cycle entry followed by G2/M arrest, possibly leading to a non-apoptotic cell death, arguably pyroptosis. Further experimental work is required to validate the processes involved in this model.

## Additional files

Additional file 1: CD4 T cell subpopulations were separated with at least 90% purity. Dot plots showing the sequential gating strategy used to analyze the frequency of CD4 T cell subpopulations. (PDF 173 kb)
Additional file 2: CD4 T_CM_ cell signature in HIV infection. Table that lists the genes that were differentially expressed by T_CM_ cells from patients with HIV, constituting the HIV T_CM_ signature. (XLS 56 kb)
Additional file 3: RMA-normalized gene expression of differentiation-related genes. Table that lists differentiation-related genes and their normalized expression values in each CD4 T cell subpopulation sample. (XLS 631 kb)
Additional file 4: Th1 polarization pathway enriched in the T_CM_ from HIV infection. Diagram displaying Th1 polarization pathway enriched in the T_CM_ cell signature in HIV infection. (PDF 366 kb)
Additional file 5: Upstream analysis of gene expression signature in T_CM_ from HIV infection. Diagram displaying the upstream analysis of molecules related with gene expression signature in T_CM_ from HIV infection. (PDF 807 kb)
Additional file 6: RT-PCR validation of T_CM_ cell signature in HIV infection. Table that lists the results of RT-PCR validation of 91 genes of T_CM_ cell signature in HIV infection. (XLS 40 kb)
Additional file 7: Expression of four reference genes used in validation by RT-PCR. Figure that shows the expression of four reference genes used in validation by RT-PCR. (PDF 130 kb)
Additional file 8: Ingenuity functional prediction analysis of the signature of T_CM_ cells in HIV infection. Tables that show the five functions reported by ingenuity functional prediction analysis (IPA) of RT-PCR validated genes of the T_CM_ cell signature in HIV infection. (PDF 166 kb)

## Abbreviations

cDNA: Complementary DNA
DAVID: Data Base for Annotation, Visualization, and Integrated Discovery
DNA: Deoxyribonucleic acid
FC: Fold change
FDR: Falser discovery rate
GSEA: Gen Set Enrichment Analysis
HIV: Human Immunodeficiency Virus
IFN: Interferon
IL: Interleukin
IPA: Ingenuity Pathway Analysis
LPS: Lipopolysaccharide
mRNA: Messenger Ribonucleic Acid
NLRs: NOD - like receptors
PBMCs: Peripheral blood mononuclear cells
PCA: Principal component analysis
PCR: Polymerase chain reaction
PNA: Peptide nucleic acid
RANTES: Regulated on activation, normal T cell expressed and secreted. Also CCR5
RMA: Robust Multiarray Average Method
RNA: Ribonucleic acid
RT-PCR: Real time polymerase chain reaction
SIV: Simian immunodeficiency virus
T_CM_: Central memory CD4 T cells
TCR: T cell receptor
T_EM_: Effector memory CD4 T cells
TLR: Toll - like receptor
T_N_: Naive CD4 T cells
TNF: Tumor necrosis factor
