# Supplementary material for: A transcriptome-based model of central memory CD4 T cell death in HIV infection
Source: BMC Genomics. 2016 Nov 22;17:956. doi: 10.1186/s12864-016-3308-8 (PMC5120471; doi:10.1186/s12864-016-3308-8)
Supplement: Additional file 1: — CD4 T cell subpopulations were separated with at least 90% purity. Dot plots showing the sequential gating strategy used to analyze the frequency of CD4 T cell subpopulations. (PDF 173 kb) [file 12864_2016_3308_MOESM1_ESM.pdf]

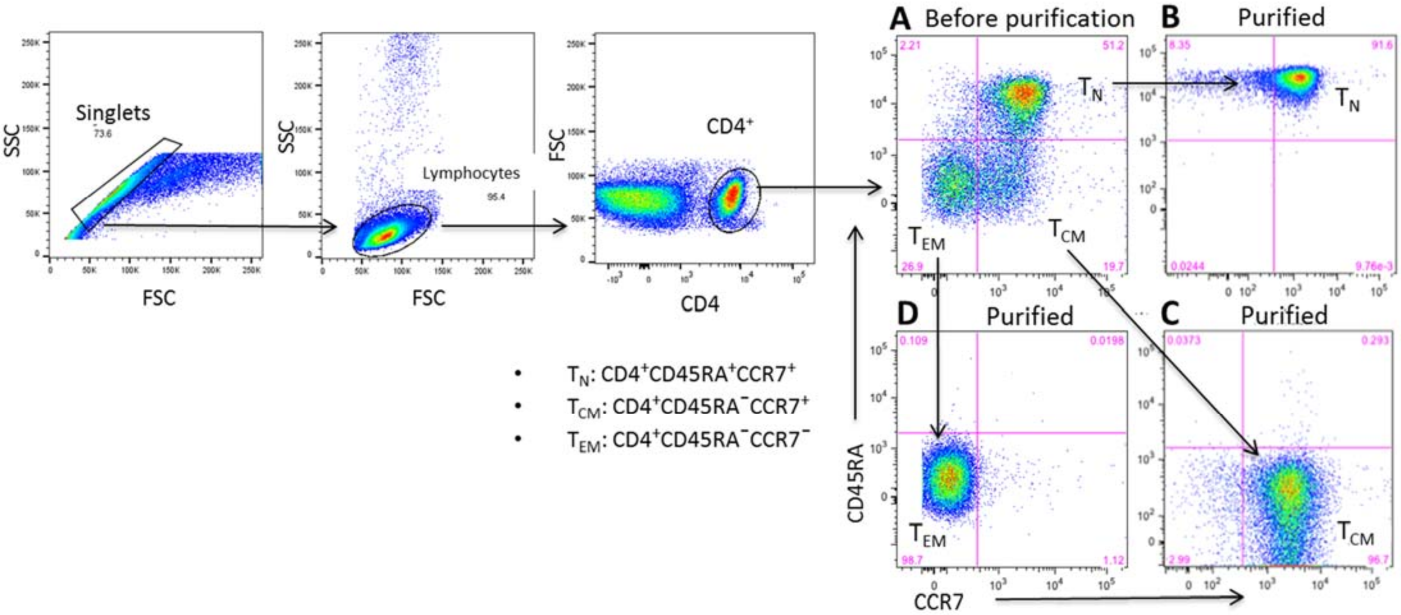

Additional file 1. **CD4 T cell subpopulations were separated with at least 90% purity**. Single CD4 lymphocytes were sub-gated into maturation subpopulations according to the expression of CCR7 and CD45RA. (A) Subpopulations among PBMCs previous to purification. (B) Purified T_N, naive CD4 T cells. (C) Purified T_{CM}, central memory CD4 T cells. (D) T_{EM}, effector memory CD4 T cells. Each subpopulation sample was obtained from a single HIV patient or healthy control with a purity of at least 90%. Gates were delineated using an FMO control for CCR7.
