# Supplementary material for: A transcriptome-based model of central memory CD4 T cell death in HIV infection
Source: BMC Genomics. 2016 Nov 22;17:956. doi: 10.1186/s12864-016-3308-8 (PMC5120471; doi:10.1186/s12864-016-3308-8)
Supplement: Additional file 5: — Upstream analysis of gene expression signature in TCM from HIV infection. Diagram displaying the upstream analysis of molecules related with gene expression signature in TCM from HIV infection. (PDF 807 kb) [file 12864_2016_3308_MOESM5_ESM.pdf]

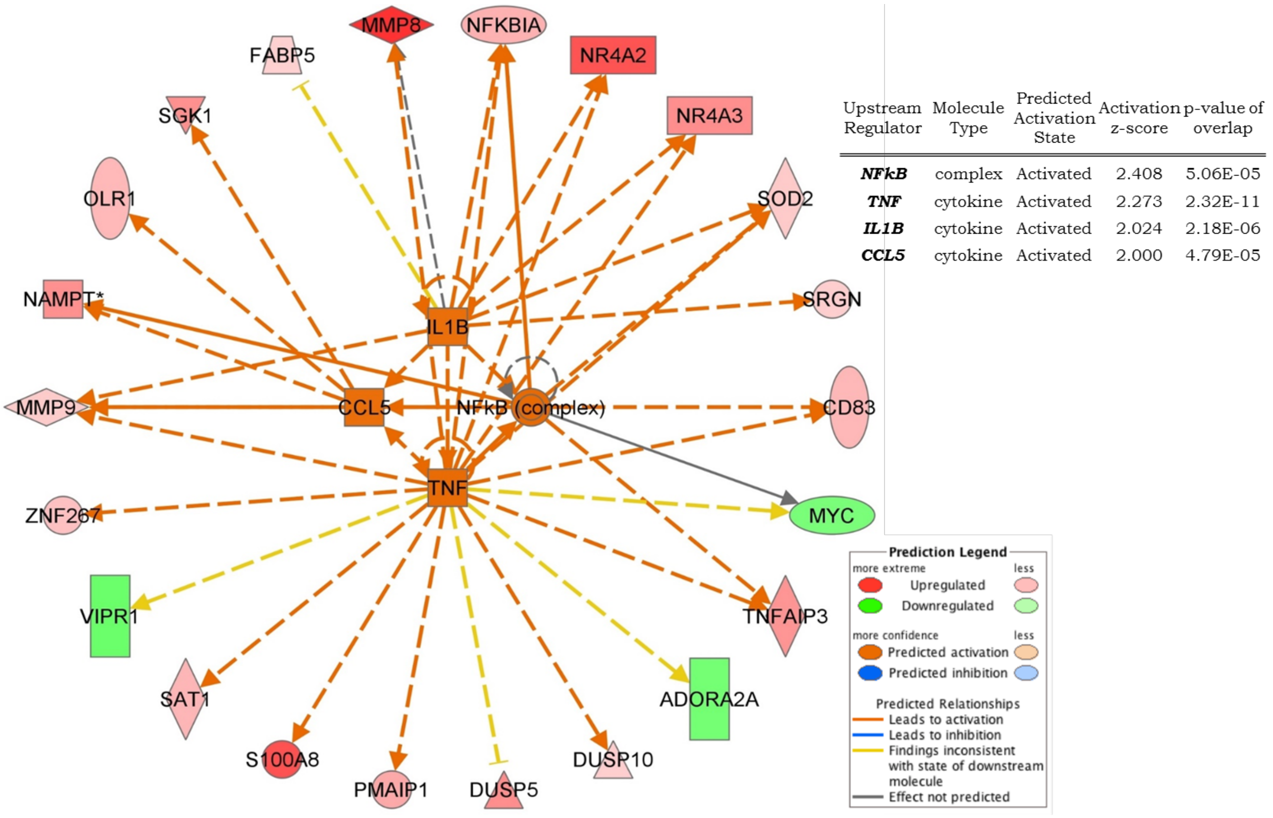

Upstream Regulator	Molecule Type	Predicted Activation State	Activation z-score	p-value of overlap
NFkB	complex	Activated	2.408	5.06E-05
TNF	cytokine	Activated	2.273	2.32E-11
IL1B	cytokine	Activated	2.024	2.18E-06
CCL5	cytokine	Activated	2.000	4.79E-05

Additional file 5. Upstream analysis of the gene expression signature of T_{CM} cells from HIV. Upstream molecules related with the gene expression signature of HIV^+ T_{CM} cells are associated with inflammatory signaling through CCL5, IL1β, TNF-α, and NFκB pathways. These upstream molecules had the highest z-score using IPA.
