# Supplementary material for: A transcriptome-based model of central memory CD4 T cell death in HIV infection
Source: BMC Genomics. 2016 Nov 22;17:956. doi: 10.1186/s12864-016-3308-8 (PMC5120471; doi:10.1186/s12864-016-3308-8)
Supplement: Additional file 7: — Expression of four reference genes used in validation by RT-PCR. Figure that shows the expression of four reference genes used in validation by RT-PCR. (PDF 130 kb) [file 12864_2016_3308_MOESM7_ESM.pdf]

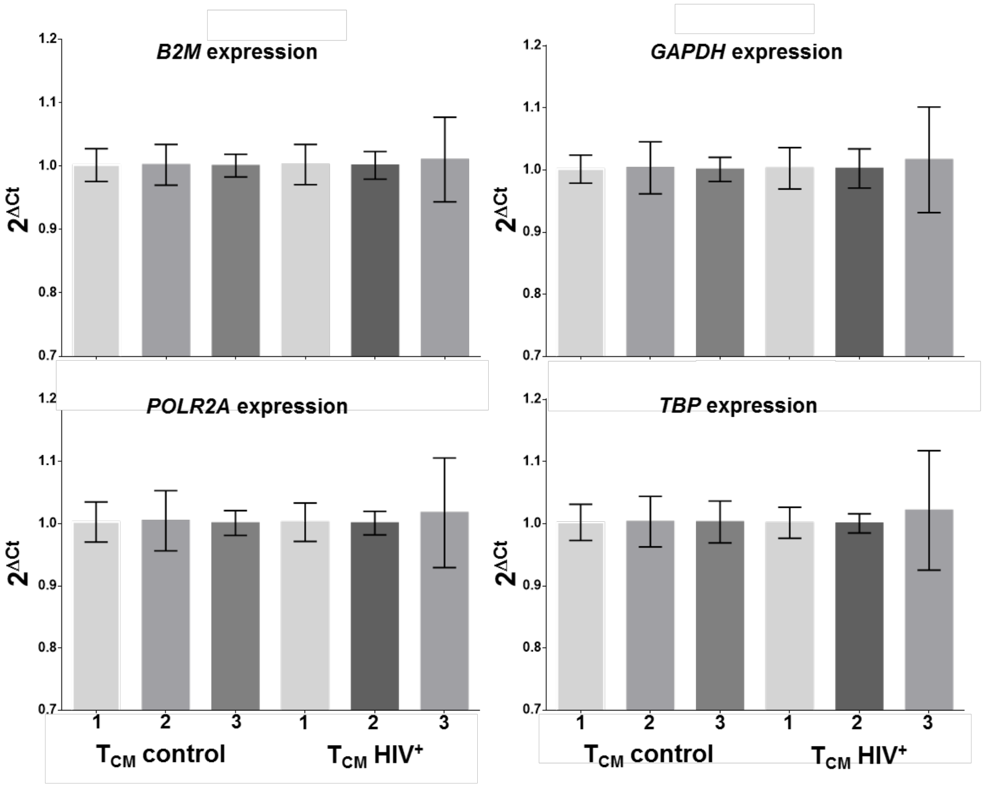

Additional file 7. Expression of four reference genes used in validation by RT-PCR. B2M, β-2 microglobulin, GAPDH, glyceraldehyde-3-phosphate dehydrogenase; POLR2A, RNA polymerase II; TBP, TATA-binding protein. Expression data of all genes was normalized with the geometric mean expression of B2M. Expression of each gene was evaluated in three six samples (1, 2 and 3 from each group), and with 6 technical replicates of each sample. Bars represent the mean value. Error bars correspond to 1 SEM. No differences were found using ANOVA.
