# Supplementary material for: A transcriptome-based model of central memory CD4 T cell death in HIV infection
Source: BMC Genomics. 2016 Nov 22;17:956. doi: 10.1186/s12864-016-3308-8 (PMC5120471; doi:10.1186/s12864-016-3308-8)
Supplement: Additional file 4: — Th1 polarization pathway enriched in the TCM from HIV infection. Diagram displaying Th1 polarization pathway enriched in the TCM cell signature in HIV infection. (PDF 366 kb) [file 12864_2016_3308_MOESM4_ESM.pdf]

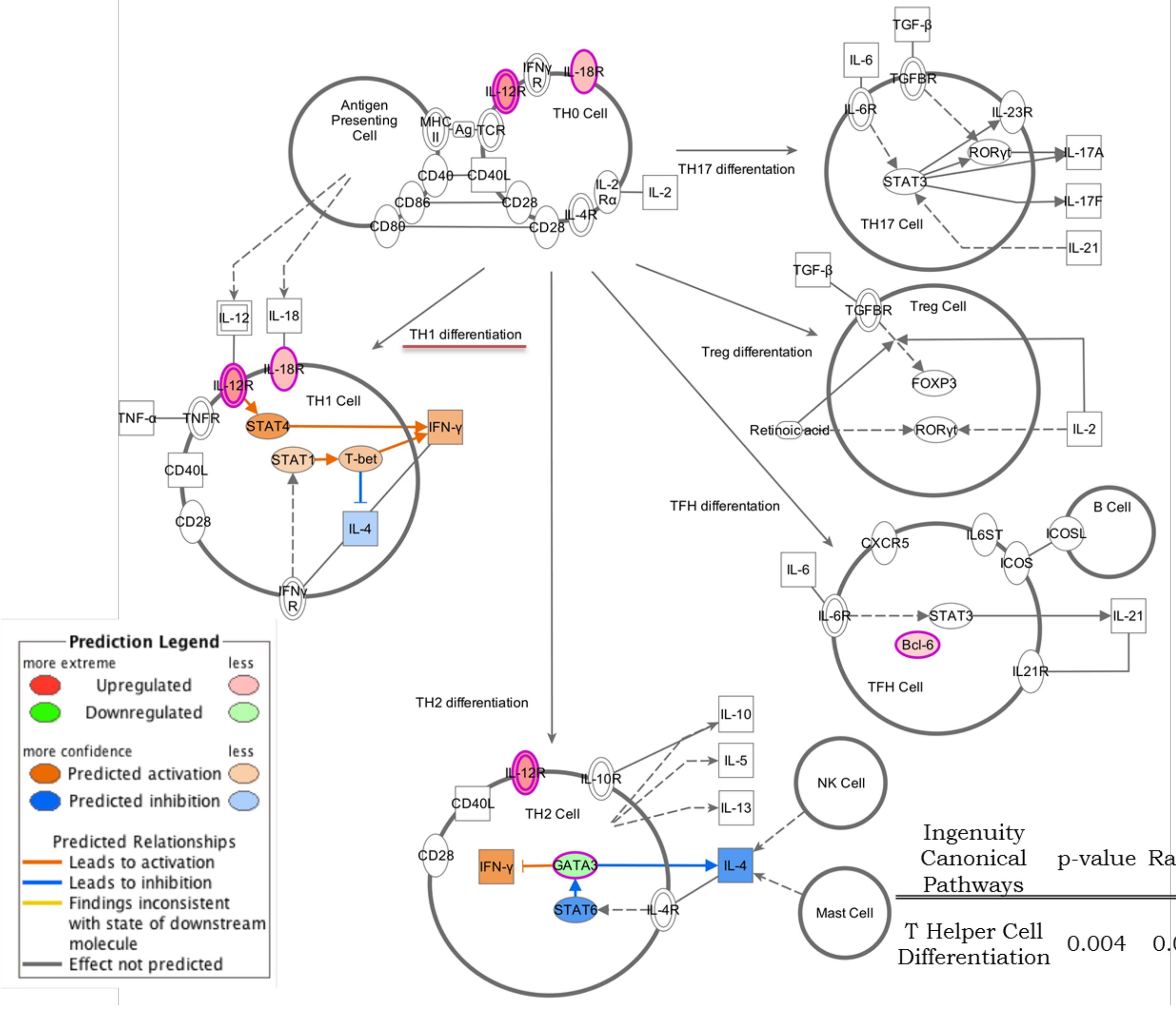

Ingenuity Canonical Pathways	p-value	Ratio	Molecules
T Helper Cell Differentiation	0.004	0.06	IL12RB2,BCL6, GATA3,IL18R1

Additional file 4. **Th1 polarization pathway is enriched in T_{CM} cells from HIV infection.** Gene expression signature of T_{CM} cells from HIV^{+} patients is consistent with a Th1-skewed functionality, according to the output of Ingenuity Canonical Pathways analysis.
