# Supplementary material for: A transcriptome-based model of central memory CD4 T cell death in HIV infection
Source: BMC Genomics. 2016 Nov 22;17:956. doi: 10.1186/s12864-016-3308-8 (PMC5120471; doi:10.1186/s12864-016-3308-8)
Supplement: Additional file 8: — Ingenuity functional prediction analysis of the signature of TCM cells in HIV infection. Tables that show the five functions reported by ingenuity functional prediction analysis (IPA) of RT-PCR validated genes of the TCM cell signature in HIV infection. (PDF 166 kb) [file 12864_2016_3308_MOESM8_ESM.pdf]

Proliferation of tumor cell lines<br>z-score = 2.7 (increased)			
Genes in dataset	Prediction	ΔΔCt	p-value
MMP9	Increased	4.960	1.20E-10
SIK1	Increased	4.219	1.05E-26
AREG	Increased	3.486	8.61E-15
ENC1	Increased	3.190	6.76E-20
PBK	Increased	2.770	1.84E-07
MNDA	Increased	2.525	2.47E-12
IL12RB2	Increased	2.364	4.83E-17
TYMS	Increased	1.889	2.47E-13
PFKFB3	Increased	1.756	2.50E-11
CD38	Increased	1.688	7.78E-15
CCNE1	Increased	1.648	3.17E-16
DTL	Increased	1.610	6.68E-09
NAMPT	Increased	1.610	3.62E-16
E2F8	Increased	1.510	9.92E-11
PER1	Increased	1.475	1.15E-11
NFKBIA	Increased	1.429	2.38E-20
MKI67	Increased	1.220	9.68E-09
PRC1	Increased	1.150	1.10E-12
CHEK1	Increased	1.110	5.31E-13
ADAM9	Increased	1.090	3.46E-08
ODC1	Increased	1.069	1.48E-14
DUSP10	Increased	1.060	2.45E-12
KIF11	Increased	0.994	4.33E-09
VAV3	Increased	0.950	1.42E-11
PTGER4	Increased	0.861	1.34E-13
ZNF267	Increased	0.791	1.27E-10
MXD1	Increased	0.670	1.40E-08
NR4A3	Increased	0.551	1.96E-03
PTP4A1	Increased	0.529	2.85E-05
NCAPG	Increased	0.334	1.62E-02
DNAJA3	Increased	-0.428	2.10E-03
FGF9	Increased	-1.980	1.72E-14
NR4A2	Increased	Upregulated*	
SGK1	Increased	NA	
TOP2A	Increased	0.230	NS
TAF1D	Increased	0.130	NS
TTK	Increased	0.090	NS
MMP8	Decreased	3.622	1.01E-14
LTF	Decreased	3.48	1.16E-11
S100A8	Decreased	3.11	5.04E-12
CD83	Decreased	2.20	6.94E-17
TNFAIP3	Decreased	2.14	1.53E-20
SAT1	Decreased	1.403	7.31E-15
DUSP5	Decreased	1.078	6.33E-09
SOD2	Decreased	0.696	9.68E-07
NRAS	Decreased	0.542	3.90E-06
MYC	Decreased	-0.946	4.04E-10
VIPR1	Decreased	NA	
HRH2	Decreased	0.07	NS
FABP5	Decreased	0.060	NS
BUB1B	Affected	1.75	1.48E-07
BCL6	Affected	1.07	1.21E-11
CD55	Affected	0.93	1.48E-09
PLK4	Affected	0.72	4.60E-06
ADCY3	Affected	-0.56	1.49E-06
ID3	Affected	NA	

Apoptosis<br>z-score=-1.9 (decreased)			
Genes in dataset	Prediction	ΔΔCt	p-value
MMP9	Decreased	4.96	1.20E-10
AREG	Decreased	3.49	8.61E-15
CSTA	Decreased	2.81	2.21E-06
PBK	Decreased	2.77	1.84E-07
TNFAIP3	Decreased	2.14	1.53E-20
TYMS	Decreased	1.89	2.47E-13
CD38	Decreased	1.69	7.78E-15
NAMPT	Decreased	1.61	3.62E-16
NFKBIA	Decreased	1.43	2.38E-20
MCM10	Decreased	1.34	4.16E-08
SRGN	Decreased	1.162	2.14E-12
CHEK1	Decreased	1.11	5.31E-13
HERPUD1	Decreased	0.99	2.05E-09
VAV3	Decreased	0.95	1.42E-11
SOD2	Decreased	0.70	9.68E-07
MXD1	Decreased	0.67	1.40E-08
NR4A3	Decreased	0.55	1.96E-03
NRAS	Decreased	0.54	3.90E-06
RNASEL	Decreased	-0.32	2.55E-02
DNAJA3	Decreased	-0.43	2.10E-03
MYC	Decreased	-0.95	4.04E-10
UBASH3A	Decreased	-1.33	2.53E-13
FGF9	Decreased	-1.98	1.72E-14
NR4A2	Decreased	Upregulated*	
SGK1	Decreased	NA	
ID3	Decreased	NA	
OLR1	Increased	3.480	5.80E-13
S100A8	Increased	3.112	5.04E-12
PMAIP1	Increased	2.640	3.10E-13
CCNE1	Increased	1.648	3.17E-16
PER1	Increased	1.475	1.15E-11
SAT1	Increased	1.403	7.31E-15
MAP3K8	Increased	1.300	3.89E-10
GADD45B	Increased	1.208	1.11E-13
BCL6	Increased	1.070	1.21E-11
CD55	Increased	0.926	1.48E-09
HSPB1	Increased	-1.439	1.92E-09
CCR8	Increased	-2.500	2.47E-15
TOP2A	Increased	0.230	NS
TNFRSF10D	Increased	0.150	NS
TTK	Increased	0.090	NS
LTF	Affected	3.484	1.16E-11
ENC1	Affected	3.190	6.76E-20
ODC1	Affected	1.069	1.48E-14
ADORA2A	Affected	-1.248	7.13E-09
AKTIP	Affected	-0.007	NS

Interphase of tumors cell lines<br>(G1/S/G2)<br>z-score=1.8 (increased)			
Genes in dataset	Prediction	ΔΔCt	p-value
CCNE1	Increased	1.65	3.17E-16
E2F8	Increased	1.51	9.92E-11
NFKBIA	Increased	1.43	2.38E-20
MCM10	Increased	1.34	4.16E-08
CHEK1	Increased	1.11	5.31E-13
RBBP8	Increased	0.80	1.01E-08
MXD1	Increased	0.67	1.40E-08
MYC	Decreased	-0.95	4.04E-10
ID3	Decreased	NA	
MMP9	Affected	4.96	1.20E-10
TYMS	Affected	1.89	2.47E-13
DTL	Affected	1.61	6.68E-09
ZBTB10	Affected	1.17	3.48E-13

Cytostasis (arrest)<br>z-score=2.2 (increased)			
Genes in dataset	Prediction	ΔΔCt	p-value
PER1	Increased	1.48	1.15E-11
NFKBIA	Increased	1.43	2.38E-20
GADD45B	Increased	1.21	1.11E-13
BCL6	Increased	1.07	1.21E-11
MXD1	Increased	0.67	1.40E-08
TOP2A	Increased	0.23	NS
NRAS	Affected	0.54	3.90E-06

Mitosis<br>z-score=-1.187 (decreased)			
Genes in dataset	Prediction	ΔΔCt	p-value
CHEK1	Decreased	1.11	5.31E-13
KIF11	Decreased	0.99	4.33E-09
RBBP8	Decreased	0.80	1.01E-08
FGF9	Decreased	-1.98	1.72E-14
BUB1B	Increased	1.75	1.48E-07
CCNE1	Increased	1.65	3.17E-16
SKA3	Affected	2.77	6.37E-04
PTP4A1	Affected	0.53	2.85E-05
TOP2A	Affected	0.23	NS
TTK	Affected	0.09	NS

Additional file 8. **Ingenuity functional prediction analysis of the signature of T_{CM} cells in HIV infection**. Ingenuity functional prediction analysis was made with IPA software. ΔΔCt and p-values represent RT-PCR validation values. * Upregulation was inferred in the absence of a ΔΔCt value, based on amplification in patients' cells (ΔCt=6.5), but no amplification in cells from controls. NS: Not significant. NA: no amplification.
